# Refinement and Modification of Al_2_O_3_ Inclusions in High-Carbon Hard Wire Steel via Rare Earth Lanthanum

**DOI:** 10.3390/ma16145070

**Published:** 2023-07-18

**Authors:** Zhoushao Ning, Changrong Li, Jie Wang, Yongqiang Zhai, Xingqiang Xiong, Lu Chen

**Affiliations:** 1 College of Materials and Metallurgy, Guizhou University, Guiyang 550025, China; nzs18893494794@163.com (Z.N.); xxq15223673790@163.com (X.X.); chen841283779@163.com (L.C.); 2 Guizhou Province Key Laboratory of Metallurgical and Process Energy Saving, Guiyang 550025, China; 3 Shougang Shuicheng Iron and Steel (Group) Co., Ltd., Liupanshui 553000, China; tianyoow@126.com (J.W.); clyyjxy520@126.com (Y.Z.)

**Keywords:** high-carbon hard wire steel, Al_2_O_3_ inclusion, inclusion modification, rare earth La

## Abstract

In this paper, an experimental protocol of adding rare earth lanthanum (La) was used to refine and modify inclusions (Al_2_O_3_) in aluminum-deoxidized steel. An optical microscope (OM), a scanning electron microscope (SEM), and an energy dispersive spectrometer (EDS) were used to study the impact of size distribution, number density, distribution uniformity, interfacial distance, area density, and so on of rare earth La on high-carbon hard wire steel inclusions. As indicated by the findings when the addition amount of La is 0.063%, the refining and homogenizing effect of Al_2_O_3_ inclusions in steel is the best. The average diameter of the inclusions is 1.75 μm, the uniformity is 0.84, the proportion of the interfacial spacing greater than 10 μm is 48.4%, and the area density of inclusions is set at 0.014. Based on classical thermodynamics and Factsage software, the effect of La activity on inclusion formation was computed. As indicated by the findings, the addition of rare earth La mainly combines with O and S in the liquid steel, and the La-containing inclusions wrap around the Al_2_O_3_ inclusions, hindering the Al_2_O_3_ inclusions. Through the evolution of inclusions during solidification, the modification of Al_2_O_3_ inclusions via rare earth La and the types of inclusions are discussed. The experimental results and theoretical calculations verify that the optimal treatment plan is to add 0.063% La.

## 1. Introduction

High-carbon hard wire steel is widely used in industry and has excellent mechanical properties [1]. The randomly distributed non-metallic inclusions in the steel matrix affect the material’s properties. Especially in the drawing of high-carbon hard wire steel, the size and morphology of non-metallic inclusions directly lead to fracture [2,3,4,5,6,7,8].

Aluminum possesses a potent deoxidizing ability and is frequently employed as a deoxidizer in the process of steel production. Aluminum and oxygen form sharp and coarse Al_2_O_3_ inclusions, which cause nozzle clogging during solidification and affect production efficiency, and large-size Al_2_O_3_ inclusions will also affect the drawing performance of the steel [9,10]. The most common modified Al_2_O_3_ inclusions are the addition of calcium and magnesium, and some rare earth. Yang et al. [11] found that MgO·Al_2_O_3_ inclusions in Al-killed steel were transformed into complex liquid inclusions via calcium treatment, and adding more calcium in molten steel would reduce the content of Al_2_O_3_ and MgO and increase the content of CaO in inclusions. Zhang et al. [12] studied the effect of magnesium addition on the composition of inclusions. The results show that the number and diameter of inclusions in all experimental steel samples are well controlled, which is helpful in improving the properties of steel. Rare earth lanthanum can reduce the content of residual elements such as oxygen, sulfur, and phosphorus in steel, prevent them from penetrating inward along the grain boundary of billet surface, and prevent the occurrence of network cracks during rolling [13]. The ability to resist cracks between inclusions and grain boundaries is improved, and the high temperature plasticity of steel materials is also greatly improved [14]. Rare earth lanthanum can play a role in modifying inclusions, improving their shape and size, making it difficult to produce large stress concentration around such rare earth inclusions, and improving the fatigue performance of steel [15]. Li et al. [16] investigated the influence of rare earth cerium on the morphology, size, and distribution of Al_2_O_3_ inclusions in low-carbon and high-manganese steels through composition analysis and theoretical calculations. The results show that Ce can change the morphology and type of Al_2_O_3_ inclusions. Irregular Al_2_O_3_ inclusions are replaced by smaller and dispersed spherical cerium oxide. Wang [17] studied the modification route of rare earth yttrium (Y) on alumina inclusions: Al_2_O_3_ → Y_2_S_3_ + YAlO_3_ + Al_2_O_3_ → Y_2_S_3_ + YAlO_3_ + Y_2_O_2_S + YAlO_3_ + Al_2_O_3_ → Y_2_S_3_ + Y_2_O_2_S. Rare earth La and Ce are lanthanide elements which have research significance for the modification of inclusions. There are many thermodynamic studies on the treatment of Al_2_O_3_ inclusions with rare earth in molten steel [18,19,20,21,22,23,24]. There are few studies on the addition of rare earth-treated Al_2_O_3_ inclusions with high-carbon steel as experimental raw material; reports on the kinetic surface in particular are limited [25,26,27]. Although there are many studies on the treatment of inclusions via calcium magnesium and Ce, Y, there are few studies on rare earth treatment. The effect of the single rare earth element lanthanum in high-carbon hard wire steel has rarely been reported on.

In this study, the effects of different La additions on the number, size, and morphology of Al_2_O_3_ inclusions were studied. The size distribution, number density, degree of homogeneity, inter-surface distance, area density, etc., of inclusions in steel under different La additions are calculated. Combined with the calculation results of Factsage, the influence of the addition of rare earth La on Al_2_O_3_ inclusions was analyzed, and the reaction types of inclusions in steel and the solidification process of inclusions in molten steel were discussed. The findings of this study provide a useful reference for addressing the modification of Al_2_O_3_ inclusions in high-carbon steel.

## 2. Experimental Methods and Procedures

The experiment involved melting the raw materials using an intermediate frequency induction furnace. The master alloy was pure iron (99.5%), recarburizer (C ≥ 98.5%, S ≤ 0.05%); Fe-Mn alloy was added to the alumina crucible of the induction furnace (size: 170 mm OD × 150 mm ID × 280 mmHT, capacity: 20 kg) for melting. For each experiment, a total of 10 kg of material was added into the crucible. Upon heating the intermediate frequency induction furnace to 1873 K, it was stirred until the steel completely melted. After ten minutes, aluminum bar (Al ≥ 98%, Si ≤ 0.6%, Fe ≤ 0.7%) was added for deoxidization and stirred with molten steel. After another ten minutes of stirring, La particles (Purity 99.9%) were added and stirred with molten steel again. After five minutes, the molten steel was poured into a mold coated with talcum powder to cool in the air. The whole experiment was divided into four groups and different amounts of rare earth La were added under the same experimental conditions in each group. Among them, the final La addition amount (calculated by 10% yield [28]) of the A1-A4 smelted steel was determined via inductively coupled plasma emission spectrometry (ICP), as shown in Table 1.

The experimental product was cut into small squares (8 mm × 8 mm × 12 mm) and ground with sandpaper from 240 mesh to 3000 mesh, then polished, washed, and dried with ethanol. Energy dispersive spectroscopy (EDS) and scanning electron microscope (SEM) were used to analyze the size, morphology, and composition distribution of inclusions. A total of 100 SEM photos were continuously shot at 1000 times magnification. A nonrepeating continuous photo with a total area of 1.7 mm × 1.038 mm was selected for each sample, and a Z-shaped movement was used when taking the sample. Image-ProPlus image processing software (Image-Pro Plus 6.0, Rockville, Media Cybernetics, MD, USA) was used to analyze the size, number, and distribution of inclusions on the sample surface. Factsage software (FACTSAGE 7.2, Thermofact/CRCT, and GTT-Technologies, Montreal, Canada, and Herzogenrath, Germany) was used, the equilibrium state of different La additions, the evolution process of inclusions, and the composition of inclusions at different temperatures were calculated, and the equilibrium state of different La additions and the evolution of inclusion composition at different temperatures were calculated. An electron probe (machine model: JXA-8530F PLUS) was used to qualitatively analyze the elements of inclusions; the acceleration voltage was 15 KV, the beam current was 10 nA, the dwell time was 15 ms, and the pixel points were collected 400 × 300.

## 3. Analysis of Results

### 3.1. Morphology and Composition of Inclusions

**Figure 1:**
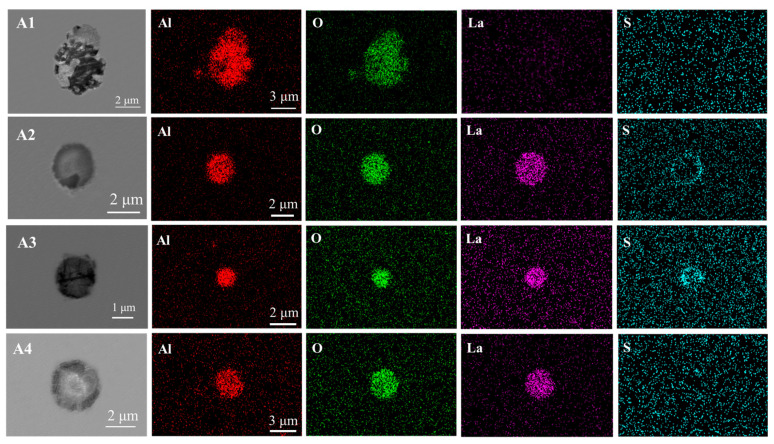
SEM/EDS image of typical inclusions in the sample A1–A4.

The morphology of the treated inclusions is shown in Figure 1. In the absence of the addition of the rare earth element La to the steel (A1), the size of Al_2_O_3_ inclusions is coarse and the shape is irregular. After adding rare earth, the inclusions are significantly refined. After modification, the content of inclusion elements mainly includes Al, O, and La, and the diameter of the inclusion is about 2.5% five μm. The two-dimensional morphology of the inclusions presents a circular shape. As can be seen from Figure 1, when the addition amount of La is 0.024% (A2), the internal oxygen and lanthanum content of the inclusion is higher, and the outer layer has less S. When the addition amount of La is 0.063% (A3), the morphology of the modified inclusion is nearly spherical, and the size of the inclusion decreases relative to the sample A2. With a 0.096% addition of La to the steel (A4), the inclusion size becomes larger relative to the sample A3, and the internal S element does not accumulate. By comparison, the inclusion size A3 < A2 < A4 < A1. This shows that the addition of trace La can change the morphology of the inclusions and modify the inclusions. The modified rare earth inclusions were flakey, spherical, and dispersed. At the same time, the adverse effects of irregular inclusions on the generation of microcracks in steel were reduced.

### 3.2. Characteristic Parameters of Inclusions

**Figure 2:**
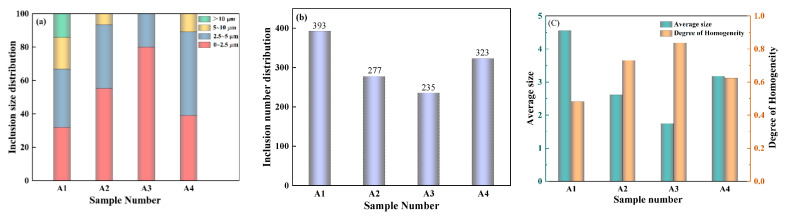
The size, number, and degree of homogeneity of inclusions. (**a**) Inclusion size distribution; (**b**) Number of inclusions; (**c**) The average size and degree of homogeneity of inclusions.

The 100 SEM images taken were imported into Image-ProPlus software to obtain various parameters of the inclusions and the parameters of the inclusions were arranged for mapping. Figure 2 showed the size distribution (a), the number distribution (b), and the distribution degree of the homogeneity of inclusions (the greater the degree of homogeneity, the more even the distribution of inclusions) (c). As shown in Figure 2a, with the addition of rare earth La, the proportion of inclusions between 0 and ~2.5 μm increases, while the ratio of inclusions exceeding 2.5 μm decreases. The greatest percentage (80%) of inclusions in A3 was found to be between 0 and 2.5 μm, and the proportions of A2 and A4 are 55.2% and 39% (both are greater than 31.8% of the A1 sample). From Figure 2b, without the addition of rare earth La, the number of inclusions was observed to be 393. After adding La, the A2 sample had 277 inclusions, while the A3 sample had 235 inclusions, and the A4 sample had 323 inclusions. Therefore, the number of inclusions decreased significantly following the addition of rare earth La. The average size of inclusions is presented in Figure 2c: A1 is 4.55 μm, A2 is 2.61 μm, A3 is 1.75 μm, and A4 is 3.18 μm; the degree of homogeneity of inclusions is as follows: A1 is 0.48, A2 is 0.73, A3 is 0.84, and A4 is 0.62. With the addition of rare earth La, the average diameter of inclusions is reduced, and the distribution is more uniform. According to the parameters of inclusions, it can be concluded that after adding La, the inclusions are refined to varying degrees. The number of inclusions is reduced, the size is reduced, and the distribution is more uniform. Among them, the A3 sample steel (with 0.063% La) has the smallest average inclusion size, the lowest number, and the most uniform distribution. Therefore, A3 sample steel has the best treatment effect, followed by A2 and A4 sample steels. Based on the analysis above, the addition of an appropriate amount of La has a refining effect on inclusions and was extremely important for the modification of Al_2_O_3_ inclusions in the steel. Dispersed fine spherical rare earth sulfur oxides replace the original Al_2_O_3_ inclusions.

**Figure 3:**
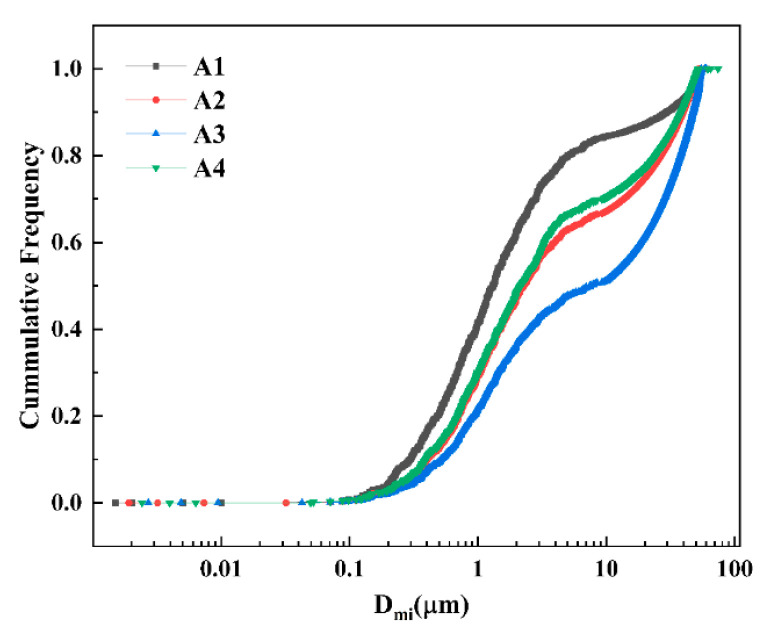
The minimum inter-surface distance distribution curve of inclusions in steel.

The inter-surface distance is calculated based on the X and Y coordinates of the inclusions in the shooting area and then counted and plotted through the pivot table (as shown in Figure 3). As can be seen in Figure 3, the proportions of the minimum inter-surface [29] distance of inclusions in A1, A2, A3, and A4 sample steels greater than 10 μm are 15.7%, 32.8%, 48.8%, and 30%. When the size of inclusions is less than 1 μm, and the minimum inter-surface distance is greater than 10 μm, the impact of inclusions on steel’s performance can be ignored [30]. A3 sample steel has the largest proportion of inclusions with a minimum inter-surface distance greater than 10 μm, followed by A2 and A4, and A1 is the smallest. This shows that the material performance of the A3 sample (with 0.063% La) is the best, the A2 sample and the A4 sample are next, and the A1 sample is the worst.

**Figure 4:**
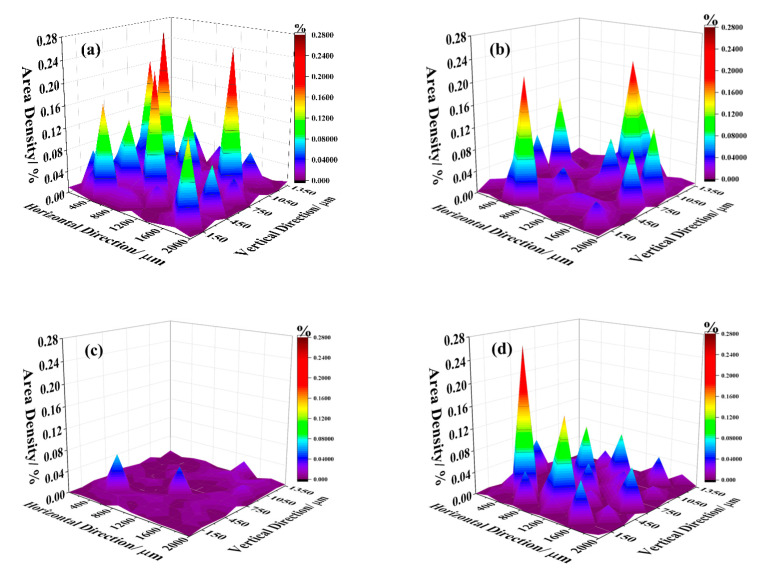
Area density distribution of inclusions on the cross section. (**a**) A1 sample; (**b**) A2 sample; (**c**) A3 sample; (**d**) A4 sample.

To provide a clearer visualization of the distribution trend of inclusions in steel, the area density distribution of inclusions was analyzed using Image-ProPlus software, as illustrated in Figure 4. From Figure 4a–d, it can be seen that the areal density of the A1 sample is concentrated at 0.08, the areal density of the A2 sample is concentrated at 0.032, the areal density of the A3 sample is concentrated at 0.014, and the areal density of the A4 sample is concentrated at 0.045. Therefore, the area density of inclusions is as follows: A3 < A2 < A4 < A1. So in the same area the A3 sample has the smallest inclusion area, which means fewer inclusions and smaller size; the A1 sample has the largest inclusion area, which means more inclusions and a larger size; the A2 sample and A4 sample were centered. The primary reason is that the A1 sample contains predominantly Al_2_O_3_ inclusions, which are not easily wetted by liquid steel, which causes inclusions to aggregate to form large-size inclusions, so the area density is large. Increasing La content in the inclusions modified Al_2_O_3_ in molten steel into small rare earth oxygen-sulfur inclusions. The interface energy of rare earth inclusions was low, the wettability with molten steel was good, and it is not easy to agglomerate, so the area density was reduced.

### 3.3. Thermodynamic Calculation

In order to determine the reaction that occurs in molten steel after adding rare earth La and the type of inclusions, the Gibbs free energy of each reaction formula was calculated. Table 2 lists the standard Gibbs free energy of each reaction formula [31].

Using the Wagner model Equation (1), the activity coefficients ($f_{i}$) for the elements [O], [S], [La], and [Al] are computed. Then, by applying the activity Equation (2), the activities ($a_{i}$) for [O], [S], [La], and [Al] are calculated, and the resulting activity values are presented in Table 3. Table 4 shows the element interaction coefficients of the experimental steel at 1873 K [32,33].

(1) $$lgf_{i}=\sum_{j}^{n}e_{i}^{j}w\left(j\%\right)$$

(2) $$a_{i}=f_{i}\cdot w\left(i\%\right)$$

**Figure 5:**
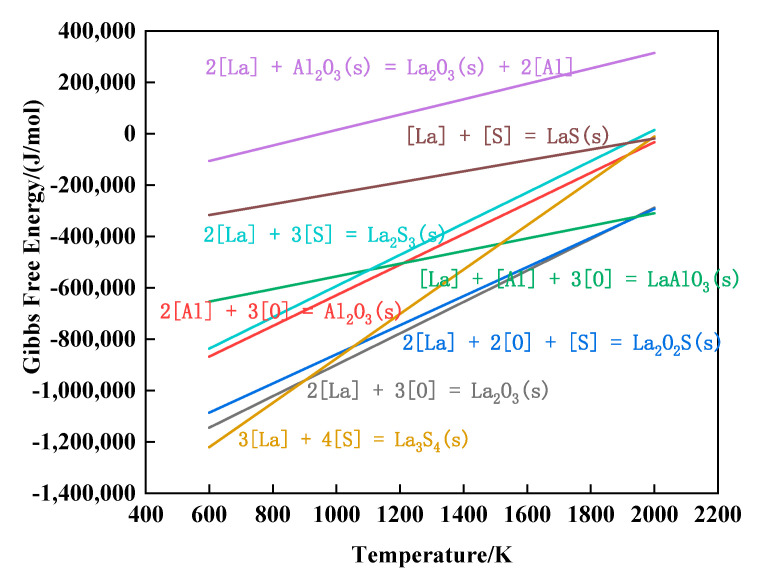
The relationship between Gibbs free energy and temperature for each reaction.

The activity calculated from Equation (2) was substituted into Equation (3) to express the K value. The relationship between the Gibbs free energy (∆G) and temperature (*T*) of each reaction formula in Table 2 can be calculated. The calculation results are shown in Figure 5.

(3) $$∆G=∆G^{\theta }+RTlnk$$

**Figure 6:**
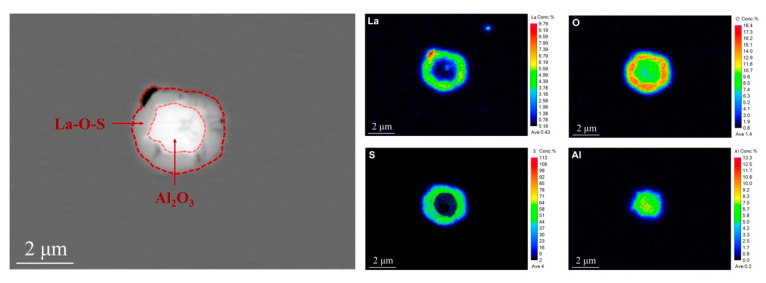
Electron Probe Detection of Inclusion Element Distribution in Experimental Steel.

According to the Gibbs free energy in Figure 5, we can confirm that when the temperature was about 1800 K (the boiling temperature of molten steel), the types of inclusions in molten steel are as follows: La-oxide, La-sulfide, La-oxysulfide, Al_2_O_3_, etc. The order of inclusions is as follows: La-oxide, La-oxysulfide, Al_2_O_3_, and La-sulfide. When the temperature is low (solidification process), [La] reacts with Al_2_O_3_ to form La_2_O_3_, which realizes the modification of Al_2_O_3_ inclusions. During the experiment, the aluminum strip was added first to deoxidize, and Al_2_O_3_ inclusions were formed. With the addition of rare earth La, La was easier to combine with [O]. La_2_O_3_ inclusions consume the surrounding oxygen during the formation process, which first satisfies the formation conditions around La_2_O_3_ inclusions, so more La-containing inclusions are produced. La-containing inclusions gradually increase, forming a circular package around Al_2_O_3_ inclusions, which hinders the accumulation and growth of Al_2_O_3_ particles, and improves inclusions and the modification of morphology, which is consistent with the description in Figure 1. In the experimental steel, an electronic probe was used for the inclusions, and it was also detected that the Al_2_O_3_ inclusions were wrapped in the center, as shown in Figure 6.

**Figure 7:**
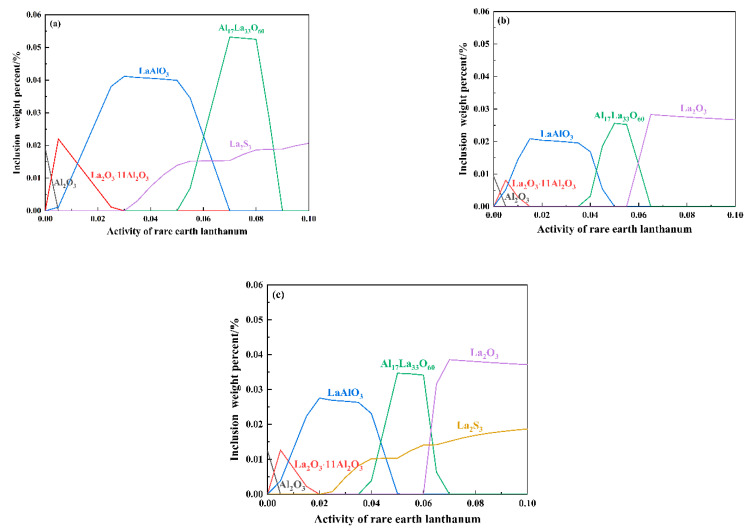
The evolution of inclusion formation at 1873 K. (**a**) A2 sample; (**b**) A3 sample; (**c**) A4 sample.

The relationship between the types of inclusions in the molten steel and the La activity needs to be calculated using Factsage software (FACTSAGE7.2, Thermfact/CRCT and GTT-Technologies, Montreal, Canada and Herzogenrath, Germany). The calculation results were shown in Figure 7.

As can be seen in Figure 7a–c, as the La content increases, the types of inclusions in molten steel are as follows: La_2_O_3_∙11Al_2_O_3_, LaAlO_3_, Al_17_La_33_O_60_, La_2_O_3_, and La_2_S_3_. From (a) to (c), it is evident that the Al_2_O_3_ content decreases, and the content of La_2_O_3_∙11Al_2_O_3_ increases, and then other inclusions are produced. In Figure 7a, the activities of [O], [S], and [Al] employed in the computation are 0.00117, 0.00855, and 0.00131. When the activity of [La] is 0.02152, La_2_O_3_∙11Al_2_O_3_ and LaAlO_3_ inclusions are formed. In Figure 7b, the activities of [O], [S], and [Al] employed in the computation are 0.00061, 0.00857, and 0.00123. When the activity of [La] is 0.05956, Al_17_La_33_O_60_ and La_2_O_3_ inclusions are formed. In Figure 7c, the activities of [O], [S], and [Al] employed in the computation are 0.00084, 0.00731, and 0.00119. When the activity of [La] is 0.08921, La_2_O_3_ and La_2_S_3_ inclusions are formed. When the addition amount of rare earth La is 0.024% (A2), the inclusions are mainly La_2_O_3_∙11Al_2_O_3_ and LaAlO_3_ (Al_2_O_3_ is also in the inclusions, and the modification is not complete). Combined with the experimental data Figure 1, Figure 2, Figure 3 and Figure 4, when the addition of rare earth La is 0.096 %, the inclusions are mainly La_2_O_3_ and La_2_S_3_, and the average size of inclusions increases to 3.18 μm, which is comparable to the average size of some original inclusions, indicating that the growth and agglomeration of inclusions are serious and the modification effect is poor. Due to the extremely high surface activity of rare earth elements, it is easy to adsorb impurity elements in molten steel. With an increase in the concentration of rare earth in molten steel, the adsorption of impurity elements also increases. Furthermore, when there is excessive rare earth, rare earth inclusions tend to attract each other, resulting in the formation of larger-sized inclusions.

Consequently, with excessive addition, rare earth inclusions were easy to agglomerate and grow [34,35,36]. Therefore, the most suitable La addition amount for the calculated inclusion type is 0.063% (A3), and the modified inclusions are mainly La oxides. Combined with the previous experimental results (the A3 experimental steel has the best effect on the modification of inclusions), the thermodynamic computations are basically in agreement with the experimental results.

### 3.4. Transformation of Inclusions during Solidification

**Figure 8:**
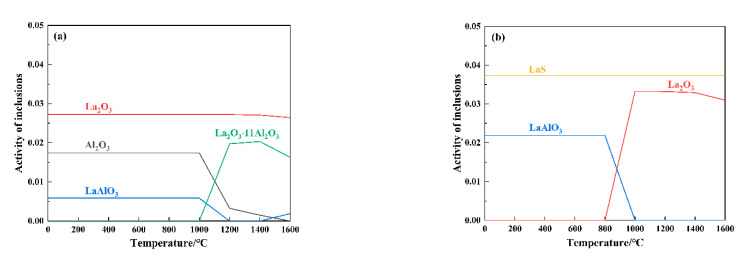

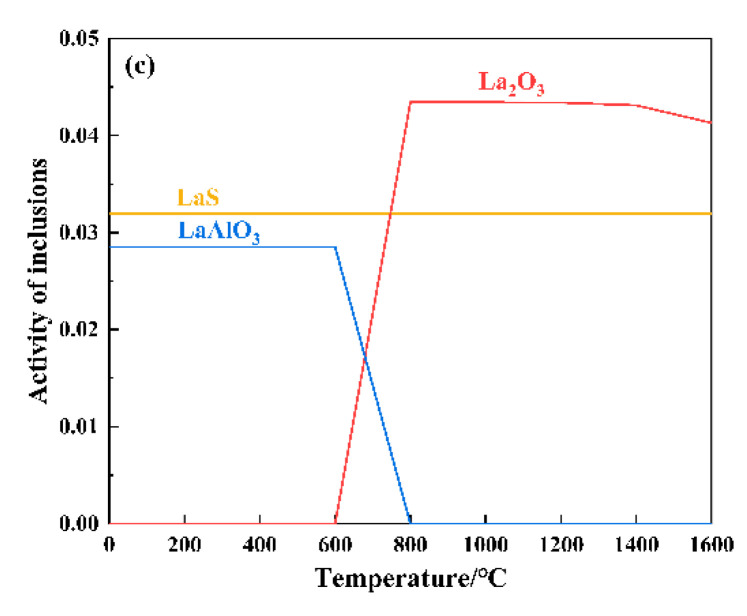
Solidification curve of inclusions in molten steel (**a**) sample A2; (**b**) sample A3; (**c**) sample A4.

According to the composition of molten steel (as shown in Table 1), Factsage software is used to calculate the equilibrium state of different La additions and the evolution of inclusion composition at different temperatures (as shown in Figure 8).

In Figure 8a, the weight percentages of [O], [S], and [Al] employed in the computation are 0.0095%, 0.007%, and 0.122%. In Figure 8b, the weight percentages of [O], [S], and [Al] employed in the computation are 0.0049%, 0.007%, and 0.116%. In Figure 8c, the weight percentages of [O], [S], and [Al] employed in the computation are 0.0064%, 0.006%, and 0.107%. In the absence of La addition to the experimental steel, the inclusions are predominantly composed of Al_2_O_3_. When 0.024% La is added to steel (as shown in Figure 8a), at 1600 °C, the inclusions are mainly composed of La_2_O_3_ and La_2_O_3_∙11Al_2_O_3_; from 1600 °C to 1400 °C, with a decrease in LaAlO_3_, La_2_O_3_∙11Al_2_O_3_ and Al_2_O_3_ increase; from 1400 °C to 1000 °C, as the temperature decreases, La_2_O_3_∙11Al_2_O_3_ decomposes and the content decreases, and the content of LaAlO_3_ and Al_2_O_3_ inclusions increases significantly; finally, the molten steel is mainly La_2_O_3_, Al_2_O_3_, and LaAlO_3_ inclusions. When 0.063% La is added to steel (as shown in Figure 8b), at 1600 °C, inclusions are mainly composed of La_2_O_3_ and LaS; from 1600 °C to 1000 °C, the steel’s inclusion content undergoes minimal alteration; from 1000 °C to 800 °C, the La_2_O_3_ content decreases and the LaAlO_3_ content increases; the final molten steel is mainly LaS and LaAlO_3_ inclusions. When 0.096% La is added to steel (as shown in Figure 8c), at 1600 °C, inclusions are mainly composed of La_2_O_3_ and LaS; from 1600 °C to 800 °C, the content of inclusions in steel changes little; from 800 °C to 600 °C, the La_2_O_3_ content decreases and the LaAlO_3_ content increases; the final molten steel is mainly LaS and LaAlO_3_ inclusions.

During the solidification of molten steel, the evolution process of inclusions is compared: when the addition amount of La is 0.024%, there are more types of inclusions (four types) after 1600 °C and cooling down, including Al_2_O_3_ inclusions, indicating that the modification is incomplete. When the addition amount of La is 0.063%, the content of La_2_O_3_ at 1600 °C is low and starts to decrease at 1000 °C, and finally there are only two inclusions; when the addition amount of La is 0.096%, the content of La_2_O_3_ is higher until 800 °C, and the final content of the two inclusions is higher. Comparing the content, type, and transition temperature of inclusions, at a La addition amount of 0.063%, the inclusion state of the steel is in an optimal condition.

## 4. Conclusions

The modification of rare earth La on Al_2_O_3_ inclusions in high-carbon hard wire steel was discussed along with an experiment and theoretical calculations. The main conclusions were as follows:

(1) By adding a suitable amount of rare earth element La, the harmful Al_2_O_3_ inclusions in high-carbon hard wire steel were modified via rare earth, which were flakey and spherical, and most of the size was refined to about 2 μm, and the distribution was more dispersed.
(2) Through the combination of thermodynamics and experiment, with the increase in rare earth La, when the addition amount of La is 0.024%, the inclusion modification is not complete; when the addition of La is 0.096%, the inclusion modification is excessive. It was basically confirmed that the optimum rare earth La addition in the experiment is 0.063%, the modification effect of inclusions is the best, and the Al_2_O_3_ inclusions are wrapped by a LaS + La_2_O_3_ ring. During the solidification process of liquid steel, the type and content of inclusions change as the temperature decreases.

## Figures and Tables

**Table 1:** Chemical composition of the test steel (weight percent/%).

Element	C	Mn	Si	P	S	Al	O	La	Bal.
A1	0.8260	0.8300	0.2100	0.0190	0.0180	0.0254	0.0150	-	98.0566
A2	0.8200	0.7900	0.2400	0.0180	0.0070	0.1220	0.0095	0.0240	97.9695
A3	0.8200	0.7800	0.2400	0.0170	0.0070	0.1160	0.0049	0.0630	97.9521
A4	0.8100	0.7800	0.2300	0.0170	0.0060	0.1070	0.0064	0.0960	97.9476

**Table 2:** Standard Gibbs free energy of each reaction.

Number	Reaction	ΔG*^θ^*/(J·mol^−1^)
1	2[Al] + 3[O] = Al_2_O_3_(s)	−1225196 + 393.78 T
2	2[La] + 3[O] = La_2_O_3_(s)	−1511520 + 379.5 T
3	2[La] + 2[O] + [S] = La_2_O_2_S(s)	−1425820 + 351.0T
4	[La] + [Al] + 3[O] = LaAlO_3_(s)	−801616 + 28.9 T
5	3[La] + 4[S] = La_3_S_4_(s)	−1738380 + 609.6 T
6	2[La] + 3[S] = La_2_S_3_(s)	−1200990 + 425.0 T
7	[La] + [S] = LaS(s)	−445180 + 141.5 T
8	2[La] + Al_2_O_3_(s) = La_2_O_3_(s) + 2[Al]	−286520 + 270.28 T

**Table 3:** Activities of [O], [S], [Al], and [La] in all steels used at 1873 K.

Number	*a*_[O]_	*a*_[S]_	*a*_[Al]_	*a*_[La]_
A2	0.00117	0.00855	0.00131	0.02152
A3	0.00061	0.00857	0.00123	0.05956
A4	0.00084	0.00731	0.00119	0.08921

**Table 4:** Interaction factors of elements to O, S, La, Al at 1873 K.

$e_{i}^{j}$	C	Mn	Si	P	S	Al	O	La
O	−0.45	−0.021	−0.131	0.07	−0.133	−3.9	−0.2	−0.57
S	0.11	−0.026	0.063	0.029	−0.028	0.035	−0.27	-
La	-	-	-	-	-	-	−4.98	-
Al	0.091	-	0.0056	-	0.03	0.045	−6.6	-

Note: *i* = O, S, La, Al; *j* = C, Mn, Si; P; S, Al, O, La.

## Data Availability

Not applicable.
